# Ecotropic viral integration site 1 promotes metastasis independent of epithelial mesenchymal transition in colon cancer cells

**DOI:** 10.1038/s41419-017-0036-1

**Published:** 2018-01-16

**Authors:** Kasturi Bala Nayak, I. S. Sajitha, T. R. Santhosh Kumar, Soumen Chakraborty

**Affiliations:** 1 Department of Gene Function and Regulation, Institute of Life Sciences Nalco Square, Bhubaneswar, Odisha India; 2 Rajiv Gandhi Centre for Biotechnology, Thiruvananthapuram, Kerala India; 3 Department of Veterinary Pathology, College of Veterinary & Animal Sciences, Wayanad, Kerala India

## Abstract

The most indecipherable component of solid cancer is the development of metastasis which accounts for more than 90% of cancer-related mortalities. A developmental program termed epithelial-mesenchymal transition (EMT) has also been shown to play a critical role in promoting metastasis in epithelium-derived solid tumors. By analyzing publicly available microarray datasets, we observed that ecotropic viral integration site 1 (EVI1) correlates negatively with SLUG, a master regulator of EMT. This correlation was found to be relevant as we demonstrated that EVI1 binds to SLUG promoter element directly through the distal set of zinc fingers and downregulates its expression. Many studies have shown that the primary role of SLUG during EMT and EMT-like processes is the regulation of cell motility in most of the cancer cells. Knockdown of EVI1 in metastatic colon cancer cell and subsequent passage through matrigel not only increased the invading capacity but also induced an EMT-like morphological feature of the cells, such as spindle-shaped appearance and led to a significant reduction in the expression of the epithelial marker, E-CADHERIN and increase in the expression of the mesenchymal marker, *N*-CADHERIN. The cells, when injected into immunocompromised mice, failed to show any metastatic foci in distant organs however the ones with EVI1, metastasized in the intraperitoneal layer and also showed multiple micro metastatic foci in the lungs and spleen. These findings suggest that in colon cancer EVI1 is dispensable for epithelial-mesenchymal transition, however, is required for metastasis.

## Introduction

Ecotropicviral integration site 1 (EVI1), an oncogenic transcription factor, is known to be associated with adverse prognosis in several hematological malignancies and some solid cancers^1–3^. The gene was originally identified as a hotspot for proviral integration in retrovirally induced murine myeloid leukemia^1^. The oncogenic potential of EVI1 was reflected by the transformation of Rat1 fibroblasts where it shows anchorage-independent growth^4^ as well as it was shown to be essential for cell proliferation and maintenance of embryonic/adult HSC and transformed leukemic cells^5^. EVI1 was reported to be overexpressed in 53% of human colorectal cancer samples, 100% of colon adenocarcinoma samples, 100% of human colon cancer cell lines and hence its presence might affect disease progression and sensitivity to chemotherapy^6^. EVI1 represses transforming growth factor (TGF) beta signaling pathway and plays a critical role in colon cancer tumor progression^6^. However, the role of EVI1 in colon cancer migration, invasion and metastasis are yet to be deciphered.

Colon cancer is the third most common malignancy, and nearly 1.4 million new cases were diagnosed in 2012 (World Cancer Research Fund International, 2012). It is well known that the tumor-initiating cells/cancer stem cells and metastasis are two critical factors that influence the survival rate of colon cancer patients. The foundation of metastasis is laid on epithelial-mesenchymal transition (EMT) which is composed of a series of events in which epithelial cells have to undergo multiple changes to assume mesenchymal phenotype, thus inducing enhanced migratory capacity, invasiveness, metastatic potential, and drug resistance^7,8^. Although some transcription factors are reported to be involved in the regulation of EMT, the most characterized are Snai1 (also known as SNAIL), Snai2 (SLUG), ZEB1, ZEB2, TWIST1, and TWIST2, all of which are eventually known to control the expression of E-CADHERIN in cancer cells^9^. Recently it was shown that overexpression of SLUG increased cellular migration, invasion and also enhanced tumor development in colon cancer cells^10^. Our present study demonstrates that EVI1 suppresses EMT by directly repressing the transcriptional activity of SLUG. Inhibition of EMT does not diminish the ability of EVI1 to form a tumor and distant metastasis in colon cancer.

## Results

### EVI1 inversely correlates with EMT related markers in colon cancer patient samples

**Fig. 1:**
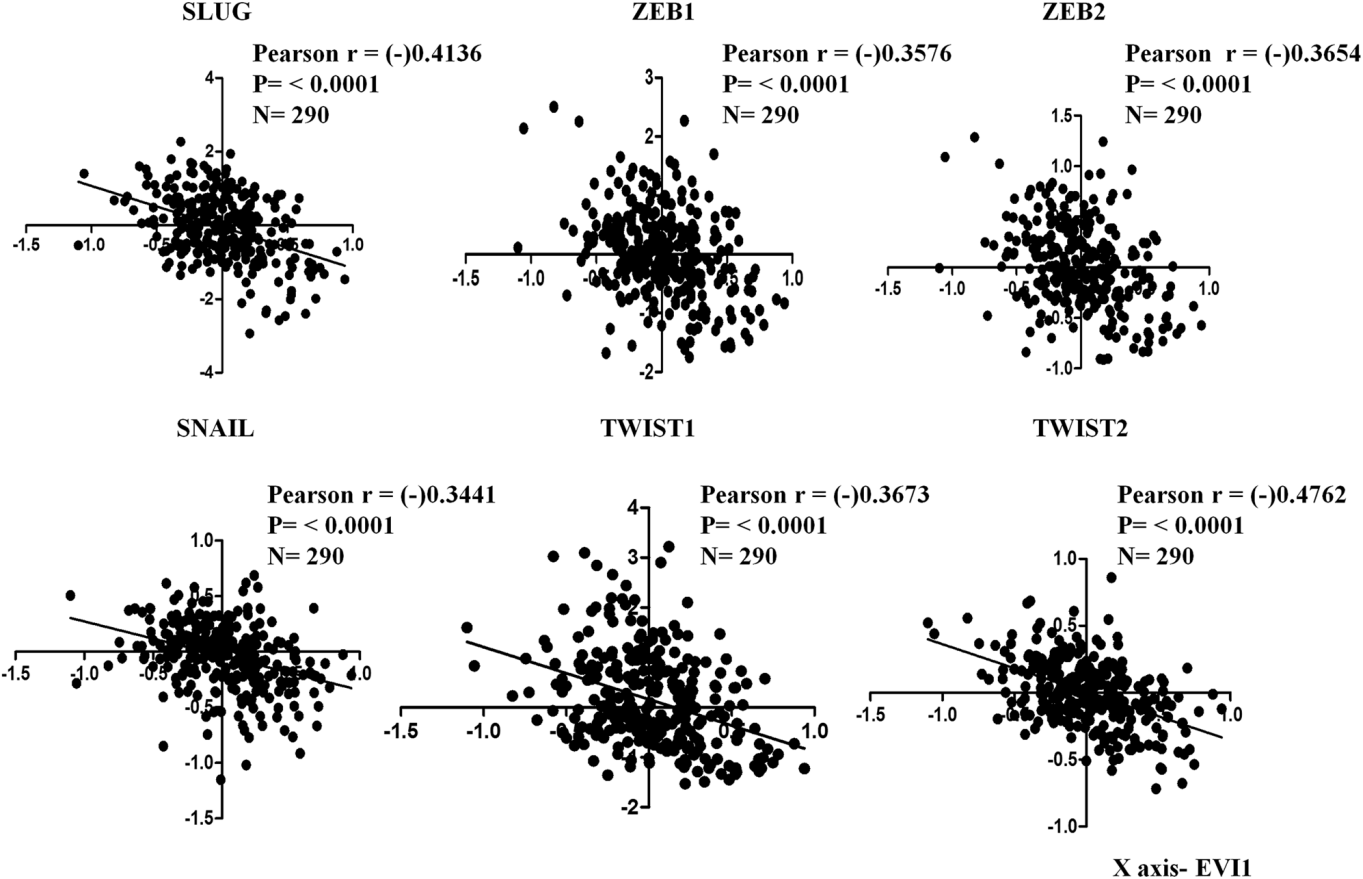
Gene expression pattern of EVI1 and correlation with EMT related genes in colon cancer Significant negative correlation was observed between the expression level of EVI1 and all the transcription factors SLUG, SNAIL, TWIST1, TWIST2, ZEB1, and ZEB2 in the GSE14333 dataset.

Earlier we have shown that EVI1 delays cell cycle progression and inhibits cell proliferation in colon cancer cells in a p53-independent manner^11^. Loss of epithelial markers and gain of mesenchymal markers play a major role to promote colon cancer cells to invade the basement membrane and the surrounding microenvironment, which eventually causes colon cancer metastasis^9^. In cancer cells loss of epithelial adhesion molecule E-CADHERIN is considered to be a fundamental event in EMT. To further investigate the role of EVI1 in colon cancer, we analyzed a colon cancer patient dataset (GSE14333) publicly available in the Gene Expression Omnibus microarray database, totaling 290 patient samples. We checked six transcription factors (SLUG, SNAIL, TWIST 1/2, ZEB1/2), all of which are known to control the expression of E-CADHERIN in cancer cells^9^. Significant negative correlation was observed between the expression level of EVI1 and all the above-mentioned transcription factors in colon cancer patient samples (GSE14333) (Fig. 1). Thus the results point to the fact that, EVI1 might play a role in regulating EMT in colon cancer.

### EVI1 directly binds to the SLUG promoter and functionally regulates its expression

**Fig. 2:**
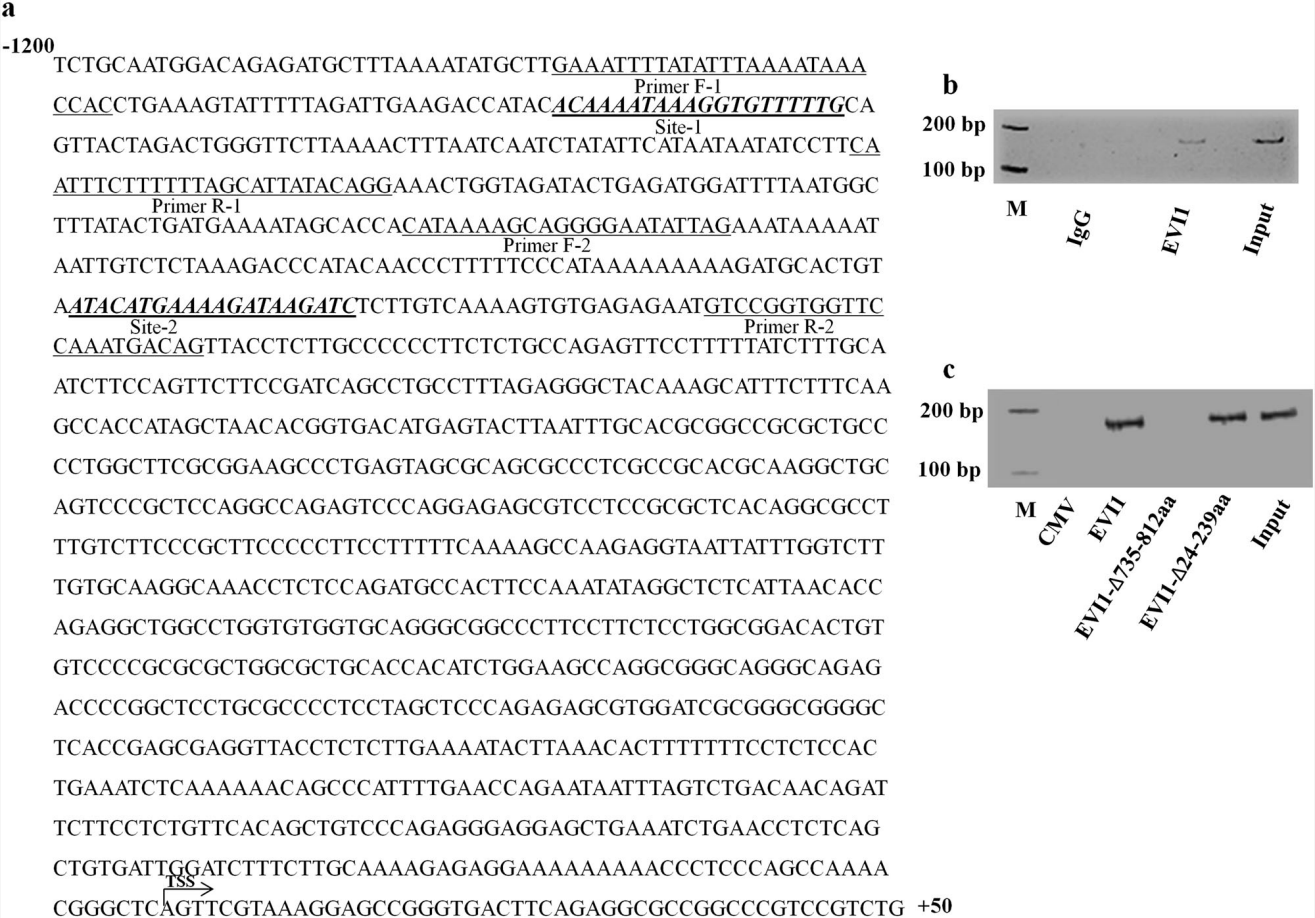
EVI1 directly binds to the SLUG promoter **a** SLUG promoter sequence extends from −1200 to +50 relative to the transcription start site (TSS) of SLUG gene. Sequences corresponding to EVI1 responsive elements (as determined by online software) are underlined as Site-1 and Site-2. The primer sequences are also underlined as primer F-1, R-1, F-2, R-2. **b** ChIP was performed on COLO205 cells, using both EVI1 and IgG antibody separately. Protein-DNA complexes were processed, and the eluted samples were analyzed by PCR. A 160 bp band was observed in the lane of EVI1, and no band was observed in IgG control. Lanes with input control and marker (M) are also shown. **c** ChIP was performed on 293T cells transfected with CMV-empty vector, flag-EVI1-wt, flag-EVI1∆24-239, and flag-EVI1∆735-812 separately. Protein-DNA complexes were processed, and eluted DNA samples were then analyzed by PCR. The 160 bp amplicon was only observed in flag-EVI1-wt and flag-EVI1∆24-239 transfected cells, but not in CMV-empty vector and flag-EVI1∆735-812 transfected cells. Lanes with input control and marker (M) are also shown.

Several emerging evidence suggests that SNAIL family proteins not only repress the E-CADHERIN expression but also induces the expression of genes associated with mesenchymal and invasive phenotypes in epithelial tumors^12,13^. To clarify the association of EVI1 with the EMT inducing transcription factors, we analyzed a recently published ChIP-sequencing data of EVI1 positive human ovarian cancer cell line SKOV3 which suggests that EVI1 might target both SLUG and SNAIL in ovarian cancer cells^14^. To understand whether EVI1 directly binds to both SLUG and SNAIL, we did an in silico analysis and searched for the EVI1 binding site(s) in the functional promoter region of both SLUG and SNAIL for almost 2 kb upstream from the transcription start site. We did not observe any binding site in SNAIL functional promoter region; however we observed two putative EVI1 binding sites with a score of 88.4 (default score 85% in TF search) in the functional promoter region of SLUG (Fig. 2a). Inspection of the human SLUG promoter in MATINSPECTOR software (www.genomatix.de/matinspector.html) showed a similar result. To validate the predicted target sites in vivo, we did chromatin immunoprecipitation (ChIP) assay in COLO205 cells. EVI1 was found to bind only to one of the SLUG binding site (site 1), out of the two predicted sites. No signal was amplified from IgG immunoprecipitated cells (Fig. 2b). To know, which DNA binding domain of EVI1, targets site-1, we used two different previously published clones of EVI1^15^, one with a deletion of the proximal set of zinc finger domain (EVI1-Δ24-239aa) and the other having deletion in the distal set of zinc finger domain (EVI1-Δ735-812aa). CMV-empty vector, flag EVI1-wt, flag EVI1-Δ24-239aa and flag EVI1-Δ735-812aa were individually transfected into 293T cells and after 48 h of transfection, ChIP assay was performed in which we found that EVI1-wt and EVI1-Δ24-239aa binds to the SLUG promoter whereas EVI1-Δ735-812aa failed to bind the promoter (Fig. 2c), which also supports the SKOV3 ChIP sequencing data^14^. All of the above data shows that EVI1 binds to site-1 identified in the SLUG promoter in vivo through the distal zinc finger domain.

**Fig. 3:**
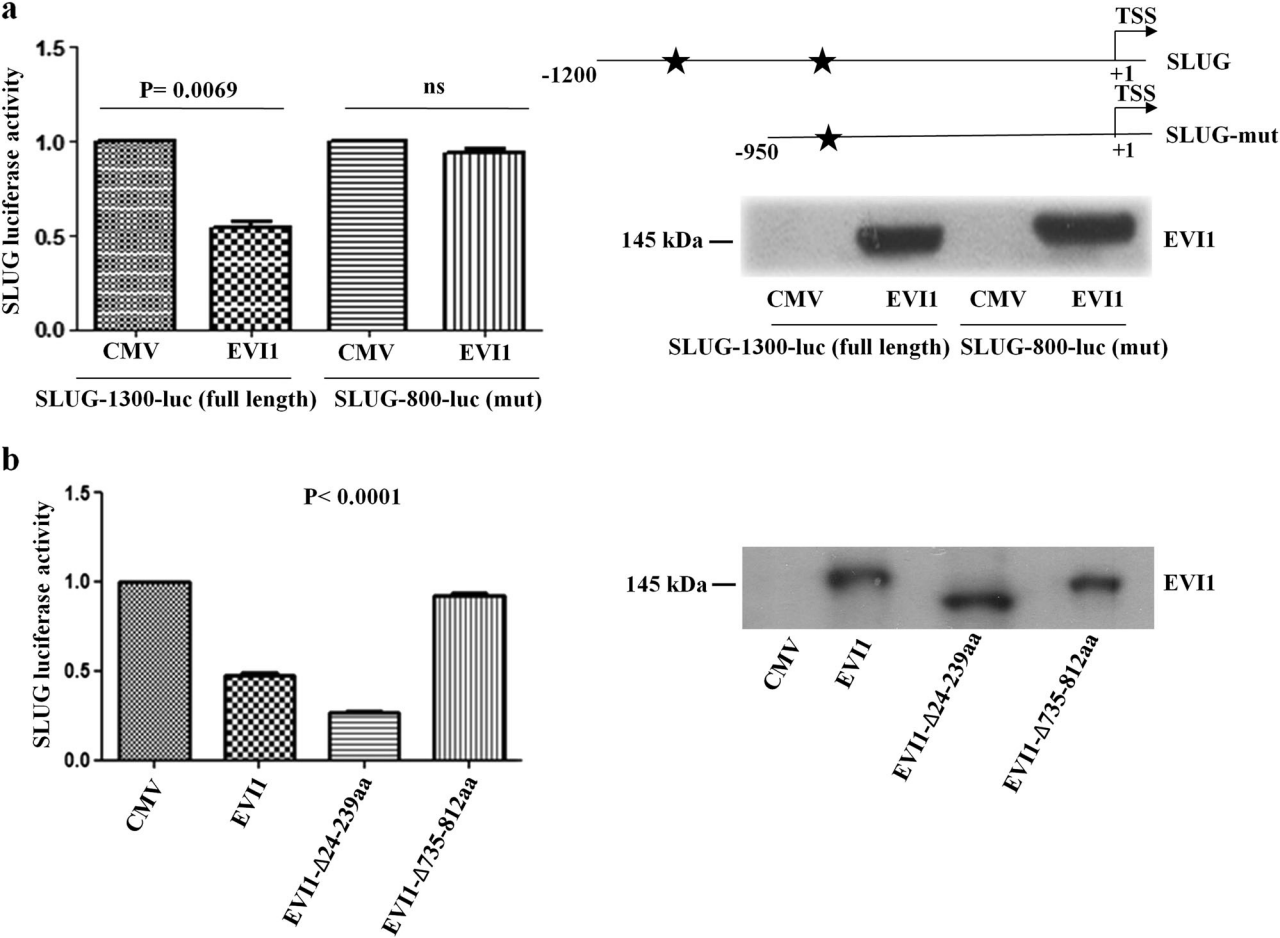
Binding of EVI1 down regulates SLUG transcriptional activity **a** 293T cells were co-transfected with CMV-empty vector or flag-EVI1-wt along with pGL3-SLUG-1200-Luc and pGL3-SLUG-950-Luc constructs (mut) and renilla luciferase as an internal control for 24 h. Data presented are fold change relative to empty vector transfected cells. Data are the means ± S.D from the triplicate analysis (Left panel). Representative expression of EVI1 is shown in the right panel. **b** 293 T cells were co-transfected with either CMV-empty vector or flag-EVI1-wt, flag-EVI1∆24-239, flag-EVI1∆735-812, along with pGL3 -SLUG-1200-Luc construct and renilla luciferase as an internal control. After twenty-four hours, luciferase activity was measured. Data presented are fold change relative to empty vector transfected cells. Data are the means ± S.D from the triplicate analysis (Left panel). Representative expression of EVI1 and the deletion constructs are shown in the right panel.

To investigate the transcriptional regulation of EVI1, we used SLUG (−1200 bp) luc construct which covers 1200 bp upstream from the SLUG transcription start site. The luciferase assay showed significantly lesser activity (approx two-fold) for EVI1 transfected 293T cells with respect to the empty vector transfected cells (Fig. 3a, bar 1 and 2). To further confirm the binding site of SLUG, we deleted the first site and made SLUG mutant luciferase construct (−950 bp). Transfection of the construct alone or in combination with EVI1 did not show any change in luciferase activity substantiating the fact that the first site is the only functional site which targets EVI1 (Fig. 3a, bar 3 and 4). We also found a significant decrease in the activity of EVI1-Δ24-239aa, whereas no such activity was observed in EVI1-Δ735-812aa (Fig. 3b). Thus all of the above data suggests that the distal zinc finger domain of EVI1 forms a complex on the promoter region of SLUG and downregulates its transcriptional activity.

### EVI1 reduces invasion capacity of colon cancer cells ex vivo

**Fig. 4:**
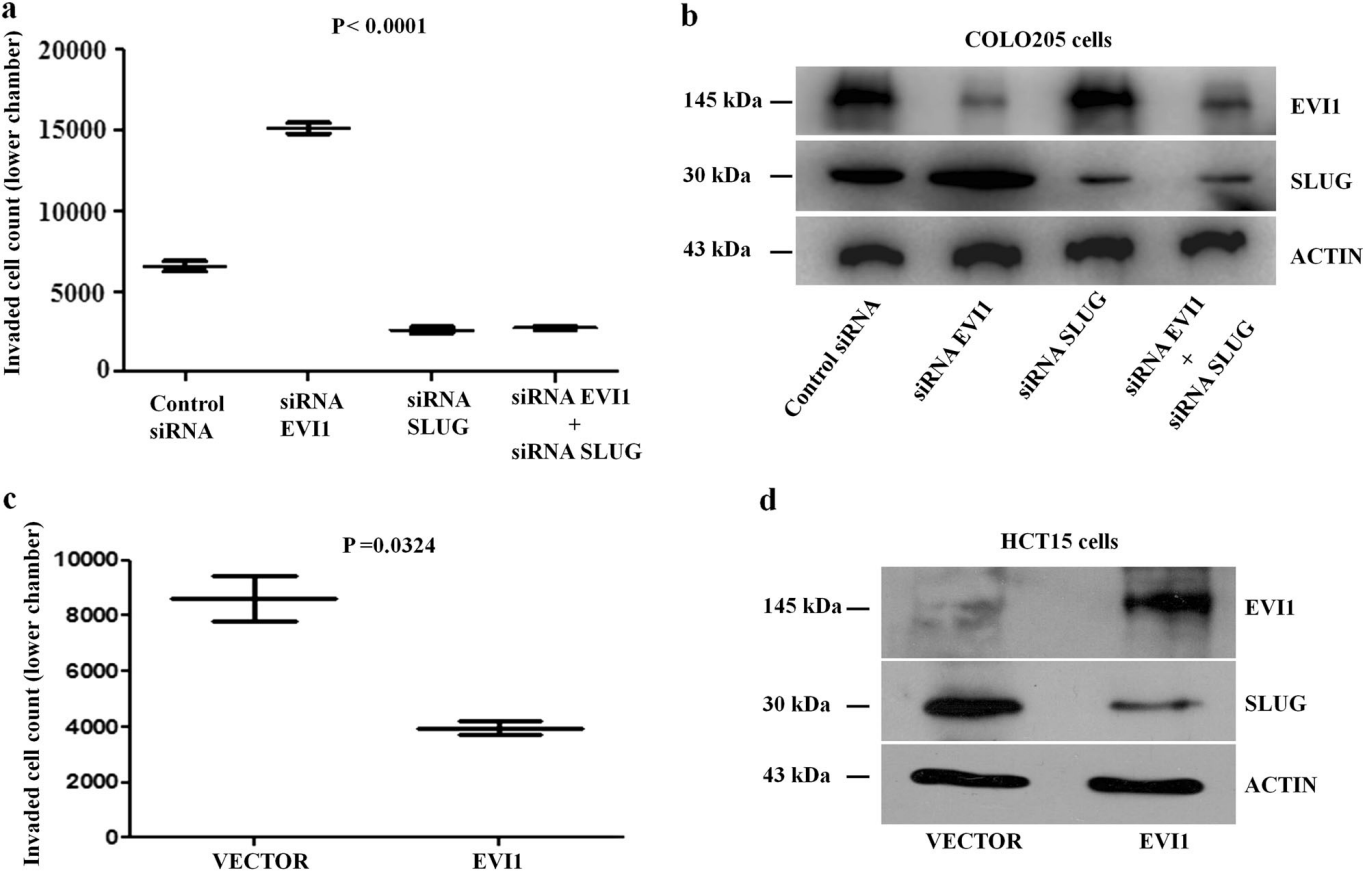
EVI1 reduces invasion capacity of colon cancer cells ex vivo **a** The cells that invaded the lower chamber of the transwell membrane were counted 48 h after plating of siRNA control, siRNA EVI1, siRNA SLUG and siRNA EVI1 + SLUG transfected COLO205 cells. The cell numbers are represented graphically. **b** The protein expression of EVI1 and SLUG in COLO205 cells after siRNA EVI1, siRNA SLUG and siRNA EVI1 + SLUG transfection are shown along with ACTIN. **c** The cells which invaded the lower chamber of the transwell membrane were counted after forty eight hours of plating of EVI1 and the empty vector transfected HCT15 cells are represented graphically. **d** The protein expression of EVI1 and SLUG in HCT15 cells after transfection are shown, along with ACTIN used as a loading control.

Several reports suggest that cancer cells undergo invasive processes and those cancer cells that have the high invasive ability are likely to cause metastasis and also show poor prognosis^16^. To understand the effect of EVI1 on cellular migration and invasion, we knocked down the expression of EVI1 alone, SLUG alone, EVI1 + SLUG in COLO205 (stage IV, metastatic) cells and did matrigel based invasion assay. Forty-eight hours after putting in transwell plate the cells in the lower chamber were counted. We observed that EVI1 siRNA transfected COLO205 cells were significantly more invasive than control siRNA, SLUG siRNA and EVI1 + SLUG siRNA transfected cells (Fig. 4a). The expression of SLUG was up-regulated with downregulation of EVI1 in COLO205 cells transfected with EVI1 siRNA (Fig. 4b). Altogether, it shows that presence of EVI1 inhibits the cell invasion capacity through SLUG.

To demonstrate the significance of EVI1 on SLUG expression, we overexpressed EVI1 in EVI1 negative HCT15 (stage III, non-metastatic) colon cancer cell line and did matrigel based invasion assay and observed that EVI1 reduces the invading capacity of HCT15 cells in comparison to empty vector transfected cells (Fig. 4c). The expression of SLUG was also found to be downregulated with EVI1 overexpression in HCT15 cells (Fig. 4d). Taken together these data substantiate the fact that presence of EVI1 down regulates SLUG and thus inhibits the invasive potential of colon cancer cells.

### EVI1 knockdown in epithelial-like COLO205 colon cancer cells induces EMT

**Fig. 5:**
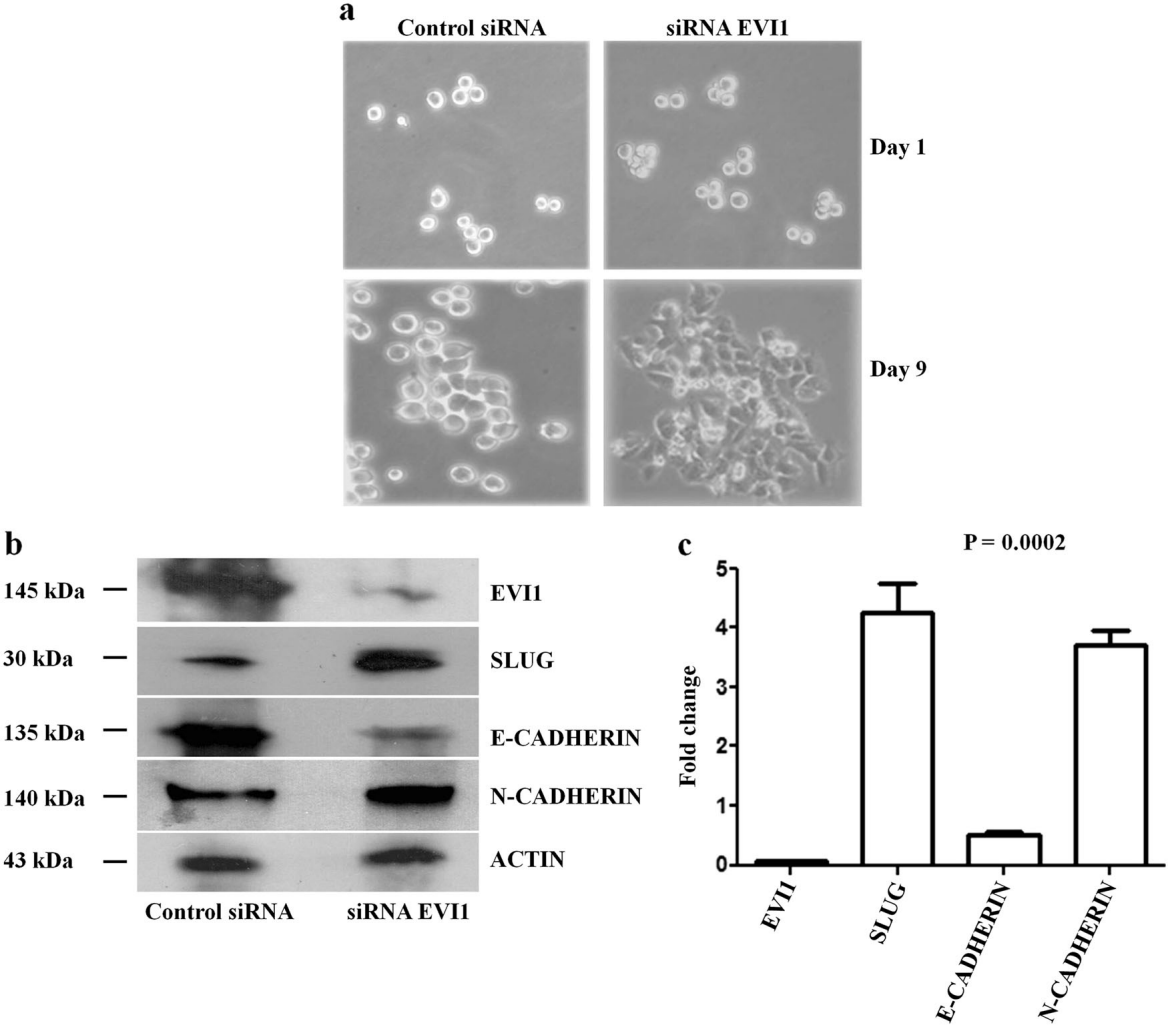
EVI1 knockdown in epithelial-like COLO205 colon cancer cells induces EMT **a** Control siRNA and siRNA EVI1 transfected COLO205 cell images after invasion are shown after 1 day (upper panel) and 9 days (lower panel). **b** The protein expression of EVI1, SLUG, E-CADHERIN, and N-CADHERIN in the EMT induced COLO205 cells are shown. ACTIN was used as a loading control. **c** RQ-PCR analysis of EVI1 and EMT-related genes in the EMT induced COLO205 cells.

EMT is known to be one of the crucial mechanism of cancer cells, where the cells are reprogrammed with more motile and invasive capacity^17,18^. Loss of EVI1 increases the invasive potential of COLO205 cells, suggesting that EVI1 may also regulate EMT related changes. To experimentally test this we used EVI1 siRNA and knocked down the expression of EVI1 in COLO205 colon cancer cells and repeated the matrigel based invasion assay. Forty-eight hours later the cells in the lower chamber were collected and cultured. After nine days of culture, we observed that EVI1 knockdown COLO205 cells induced EMT like morphological features, such as spindle-shaped appearance while control siRNA transfected cells retain their epithelial features (Fig. 5a). However, we did not observe any morphological changes in EVI1 overexpressed HCT15 cells (data not shown). To evaluate further, we checked the protein and mRNA expression of the epithelial marker E-CADHERIN and mesenchymal marker N-CADHERIN in the transformed COLO205 cells and found a significant reduction in E-CADHERIN expression and increase in N-CADHERIN expression in EVI1 knockdown COLO205 cells in comparison to the control cells (Fig. 5b, c). Here we observed that after passing through the matrigel, the cells acquire higher adherence ability than the control low adherent COLO205 cells. From the results of RT-PCR and western blot analysis, it seems that siRNA EVI1 transfected cells fail to gain the expression of EVI1 after passing through the matrigel and thus stably suppress the expression of EVI1 in EMT induced COLO205 cells. Further studies are required to understand the effect of EMT on siRNA mediated EVI1 gene silencing. We also checked for morphological changes of control siRNA treated, and siRNA EVI1 treated COLO205 cells before passing through the matrigel for 9 days. However, we did not notice any morphological changes. Collectively, these data suggest that EVI1 enforces an epithelial phenotype and acts as an inhibitor of EMT in colon cancer cells. No change in EMT was observed in EVI1 positive colon carcinoma cells like HT-29. Altogether, these data accentuate the fact that EMT in colon cancer might depend on distinct cell types. We further characterized the two different cell types to understand their biological changes.

### Induction of EMT inhibits tumor-initiating capacity in COLO205 colon cancer cell line

**Fig. 6:**
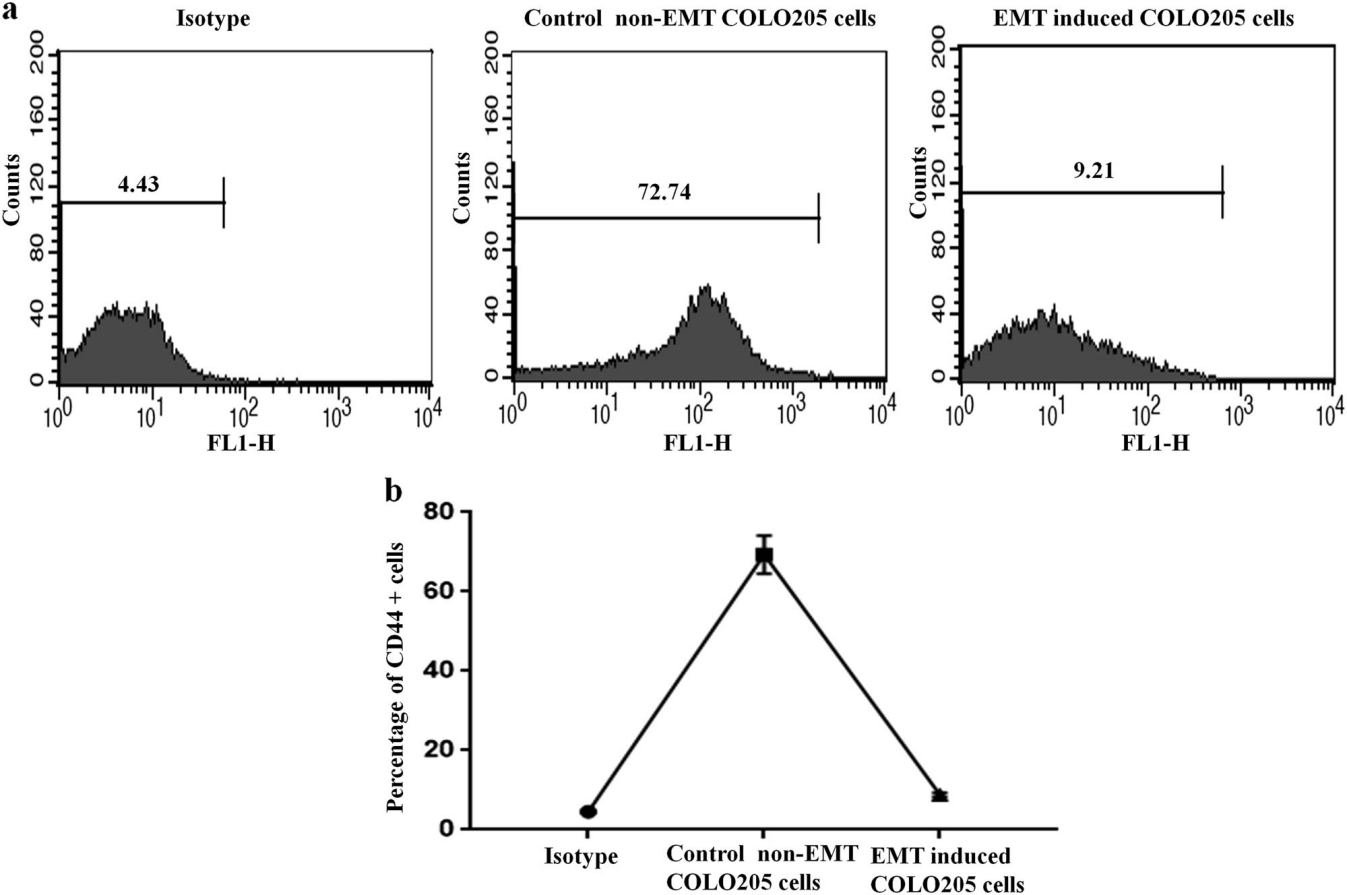
Induction of EMT inhibits tumor-initiating capacity in COLO205 colon cancer cell line **a** FACS analysis showed the expression of CD44 in control non-EMT and EMT induced COLO205 cells with respect to the isotype control. **b** The quantitative figure of the percentage of CD44+ cells in control non-EMT and EMT induced COLO205 cells with respect to the isotype control. Data were derived from two to three independent experiments.

Several reports suggest that aberrant activation of the EMT contributes to tumor initiation, progression, and therapeutic resistance^17,19–21^. Here in this study, we attempted to investigate whether EMT directly leads to the acquisition of tumor-initiating capacity of colon cancer cell lines. Du et al., 2008 reported that CD44 is a robust marker and CD44^+^ colon cancer cells are more tumorigenic than CD44^−^ and CD133^+^ colon cancer cells^22^. In some independent reports, it was shown that COLO205 colon cancer cell lines highly express CD44 stem cell marker. In benign prostatic glands, EVI1 showed exclusive expression in the basal cell layer co-localizing with CD44, which is expressed by putative stem cells^23^. We investigated the tumor-initiating capacity of the two types of cells that underwent EMT process and checked the expression pattern of CD44. In flow cytometric assay, we observed that the percentage of CD44^+^ cells are significantly decreased in the EMT induced COLO205 cells in comparison to the control non-EMT COLO205 cells (Fig. 6). Our results indicate that induction of EMT does not lead to the enhancement of tumor-initiating capacity in COLO205 colon cancer cell line. Collectively these data indicate that EVI1 might increase the capacity of cancer cells to initiate and propagate tumors upon transplantation into immuno-deficient mice.

### EVI1 supports metastasis independent of EMT in in vivo mice model

**Fig. 7:**
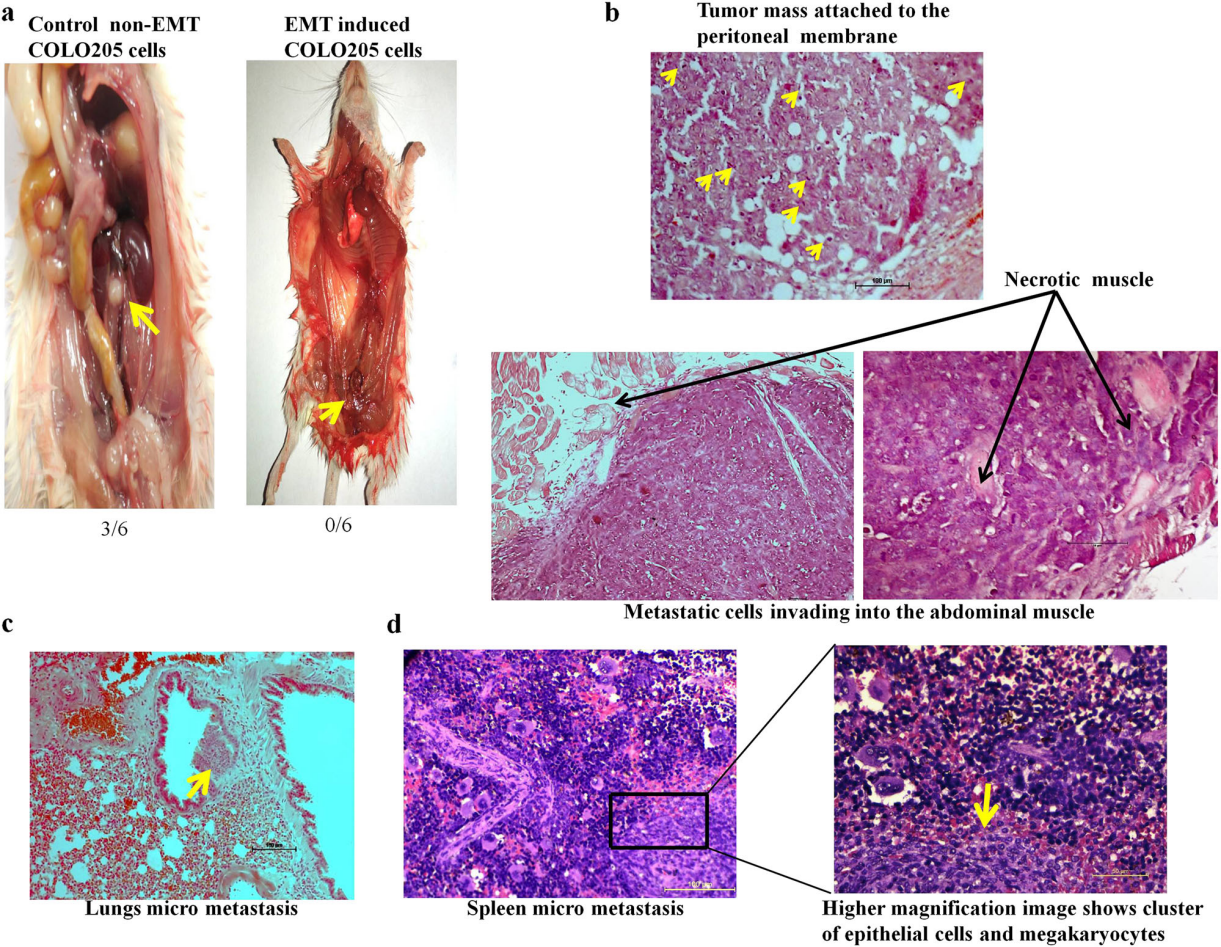
EVI1 supports metastasis independent of EMT in in vivo mice model **a** Representative macroscopic picture of the abdominal cavity of the mice injected with control non-EMT COLO205 cells, and EMT induced COLO205 cells. Metastatic nodule in the peritoneal membrane was observed in 3 out of 6 mice injected with control non-EMT COLO205 cells. However, no visible or microscopic metastasis was observed in any organ of the six mice injected with EMT induced COLO205 cells. **b** A panel of histological sections of the metastasis in the peritoneum stained with H&E. The metastatic cells were found to invade into the abdominal muscle, evident by necrosis of myocytes seen both in the periphery and inside of the growing tumor. The high proliferation potential of the cells was evident by the increased number of mitotic figures (as shown by arrows). **c** Micro metastasis was observed in the lung (as shown by the arrow). Metastatic cells were seen colonizing in the bronchial mucosa along with the inflammatory cells. **d** Micro metastasis in the spleen. A large number of mature and immature megakaryocytes were seen around the metastatic cells and the blood vessels (as shown by arrows). Higher magnification of the image showed micro metastases (as shown by the arrow) with depletion of the red pulp.

Metastasis is a highly complex and multi-step event. The general hypothesis of metastasis suggests that some cancer cells initially undergo EMT and then metastasize following clonal expansion. However, contrary to the hypothesis, recently there are several independent reports which show that forced expression of EMT in tumor cells enhanced invasiveness but inhibits their metastasis capacity^24^. SNAIL or TWIST induced EMT is not rate limiting for invasion and metastasis but important for chemotherapy treatment in pancreatic cancer^25^. Studies have also shown that overexpression of miR-200 inhibits EMT but enhance metastasis in breast cancer^26,27^. So the accumulation of all these evidence suggests that cancer heterogeneity is such that a subset of cells undergoes EMT, but it is not mandatory that all cells that have undergone EMT will successfully metastasize to distant organs. To further explore the impact of EMT on metastasis formation in vivo, we injected non-EMT COLO205 cells, and EMT induced COLO205 cells into the tail vein of immunocompromised SCID mice and observed for 4 weeks. After 4 weeks mice were sacrificed. We failed to observe any visible or microscopic colonization/ metastatic foci in any organ of the mice injected with EMT induced COLO205 cells. However, we observed that EVI1 positive non-EMT COLO205 cells consistently colonized in the peritoneal wall (Fig. 7a). A highly aggressive tumor characterized by the accumulation of pleomorphic cells with a large number of mitotic figures were seen (Fig. 7b). Multiple micro metastatic foci in lungs (Fig. 7c) and spleen (Fig. 7d) were also observed. Thus it seems that EVI1 has no effect on EMT but is an important factor for colon cancer metastasis.

### EVI1 expression correlates with E-CADHERIN, N-CADHERIN, and CD44 expression in colon cancer patient samples

**Fig. 8:**
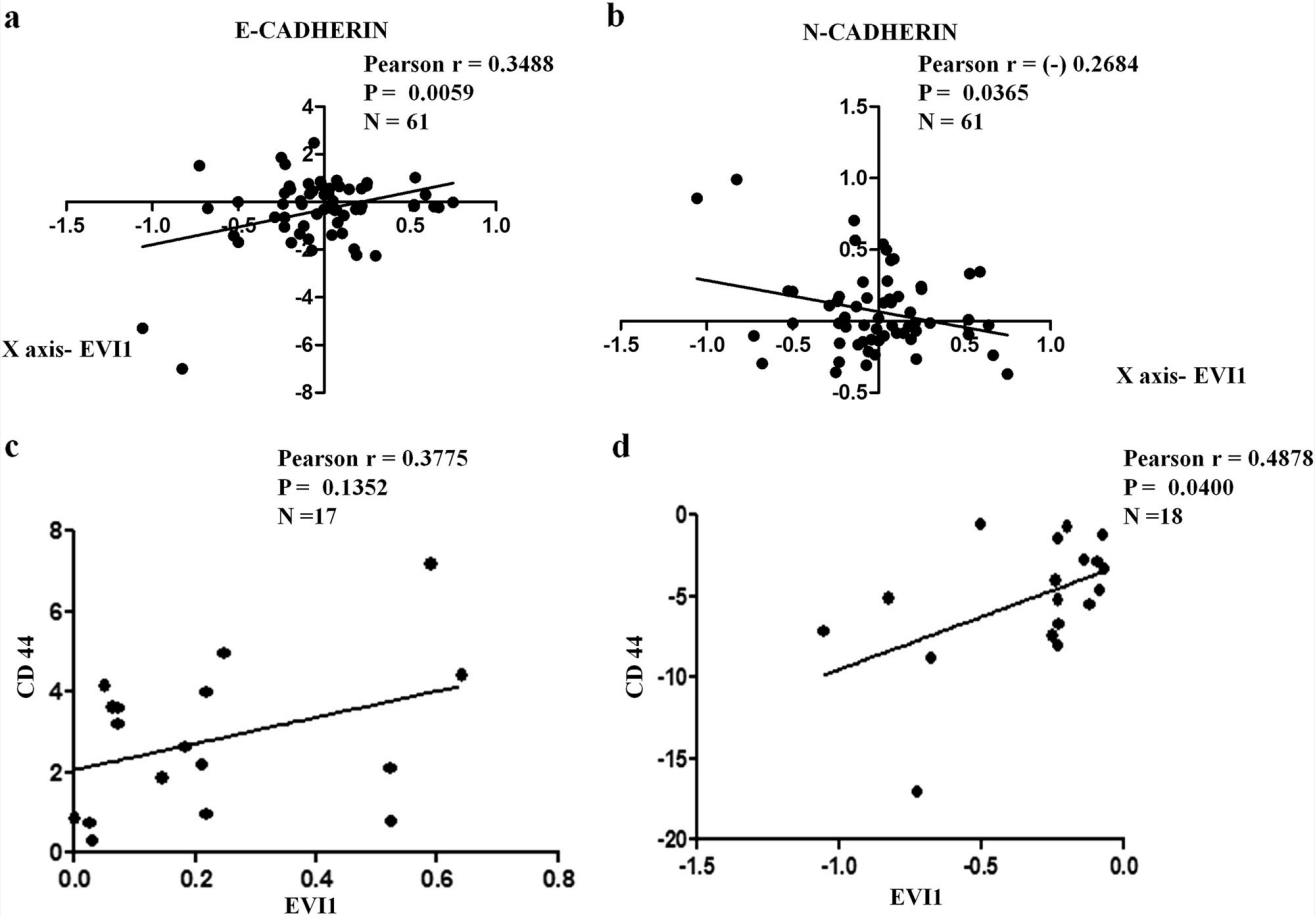
EVI1 expression correlates with E-CADHERIN, N-CADHERIN, and CD44 expression in colon cancer patient samples **a** A positive correlation between the levels of expression of EVI1 and E-CADHERIN was observed for stage IV samples (*n* = 61). **b** A negative correlation between the levels of expression of EVI1 and N-CADHERIN was observed for stage IV samples (*n* = 61). **c** A positive correlation between the levels of expression of EVI1 and CD44 was observed for stage IV samples that showed both EVI1^+^/CD44^+^ samples (*n* = 17). **d** A positive correlation between the levels of expression of EVI1 and CD44 was observed for stage IV samples that showed both EVI1^−^/CD44^−^samples (*n* = 18).

To investigate whether our experimental findings are relevant to the human colon cancer patient samples, we analyzed only stage IV patient samples from the GSE14333 dataset because cells at this stage can metastasize to other organs. Overall, we observed a positive correlation between the expression levels of EVI1 and E-CADHERIN (Fig. 8a) and a negative correlation between the expression levels of EVI1 and N-CADHERIN (Fig. 8b). Further to understand the correlation between EVI1 and CD44 in stage IV patient samples, we divided the samples into two groups, i.e., one expressing high levels of EVI1 and the other expressing low levels of EVI1. A positive correlation was observed between EVI1 and CD44 in EVI1^+^/CD44^+^ (*n* = 17) patients (Fig. 8c). Also, a strong positive correlation was observed between EVI1 and CD44 in EVI1^−^/CD44^−^ (*n* = 18) patients (Fig. 8d). However, we did not observe any significant correlation between the expression levels of EVI1 and CD44 in both EVI1^+^/CD44^−^ (*n* = 13) and EVI1^−^/CD44^+^ (*n* = 13) group of patients. Overall, the clinical data supports our observation which shows that EVI1 can act both ways; as a suppressor of EMT and also as an enhancer of metastasis in colon cancer.

## Discussion

Cancer stem cells and EMT are considered to be the two crucial factors for solid cancer biology. The capacity of initiating a tumor is a unique characteristic of a cell with stemness properties. Despite the presence of EVI1 in 100% of human colon cancer patient samples, little is known about the cofactors that are potentially regulated by EVI1. In this study by using publicly available microarray datasets, we for the first time showed a negative correlation between EVI1 and all the known EMT related transcription factors (SNAIL, SLUG, ZEB1, ZEB2, TWIST1, and TWIST2) in colon cancer patient samples. EMT is a process mediated by a set of transcription factors, with SLUG as the prominent player. SLUG is a zinc finger transcriptional repressor of the SNAIL family that promotes carcinoma cell invasion, stemness, and survival. We went on to show that EVI1 directly binds to the SLUG promoter element and functionally downregulates its transcriptional activity through its distal zinc finger domain. Transcription factor Elf5 inhibits invasion and suppresses EMT through direct transcriptional repression of SLUG in both the mammary gland epithelium and in breast cancer^28^. Many studies have supported the importance of TGFβ signaling for the repression of epithelial genes and induction of mesenchymal genes in vitro and in vivo^29,30^. Previously it was shown that EVI1 represses TGF-beta signaling by inhibiting Smad3^31^ however later it was shown that it does not affect TGF-beta mediated EMT in intestinal epithelial cells^32^. Furthermore, it was also shown that depletion of EVI1 variants leads to increased claudin-1 expression, which leads to alterations in EMT marker expression that modulates cellular motility in both breast cancer and ovarian cancer cells^33^. Silencing of EVI1 not only increased the invading capacity but also induced an EMT-like morphological feature of the cells, such as spindle-shaped appearance, and led to a significant reduction in the expression of the epithelial marker, E-CADHERIN and an increased expression of mesenchymal cell marker N-CADHERIN. Also forced expression of EVI1 in HCT15 cell line reduces its invasive capacity with respect to empty vector transfected HCT15 cells.

Several studies have suggested that the activation of the EMT contributes to tumor initiation capacity in different cancers^17,19–21^. Breast cancer is the first human tumor from which CD44^+^/CD24^−/low^ tumor initiating cells were identified and isolated^34^. These cells can self-renew, express “stemness” genes, and carry high tumorigenic and metastatic potentials^19,24^. Earlier it was speculated that an aberrant activation of stem cell factors such as EVI1 might contribute to prostate cancer CSC formation and thus promote prostate cancer initiation^26^. We found that the percentage of CD44^+^ cells are more in non-EMT COLO205 epithelial cells in comparison to EMT induced COLO205 cells. The suppression of tumor-initiating cells by EMT induced cells contradict some studies where it suggests that EMT can increase the tumor-initiating capacity of transformed cells which can enhance tumorigenic and metastatic properties^17,21^. However, our findings support other models in which it was shown that EMT and tumor initiating potential of cells are negatively related to each other^24,35,36^. These results suggest that the transition from the epithelial phenotype to the mesenchymal phenotype lead to inhibition or loss of tumor-initiating capacity in COLO205 colon cancer cells and one of the pivotal factors seems to be EVI1. Thus it seems that the tumor-initiating capacity of colon cancer cells may be independent of their mesenchymal properties.

Accumulation of data from some recent studies demonstrated that EVI1 promotes tumorigenesis and its expression correlates with metastasis and poor prognosis in breast cancer cells^33,37^. In metastatic mice model, we observed tumor nodules and lungs/spleen micro metastases induced by non-EMT COLO205 cells, contradicting the original EMT/MET hypothesis. Our data add to the growing evidence that indicates that tumor cells disseminate and metastasize while persisting in their epithelial phenotype^24–27^. Our study also shows that EVI1 is necessary for metastasis and it is not indispensable for EMT. Finally, analysis of the gene expression in stage IV of colon cancer showed a strong correlation between EVI1 and E-CADHERIN, N-CADHERIN and CD44. We showed that absence or presence of EVI1 dictates stage IV cells to either go for EMT or metastasis but not both of them. A future challenge will be to identify the entire cascade of EVI1 targets that potentiates colon cancer metastasis. The system level deregulation of EMT is important not only to understand tumor progression and the metastatic process but also to develop therapeutic interventions to halt/suppress metastatic disease.

## Materials and methods

### Cell culture

Human colon cancer cell lines COLO205, HT29, HCT15 and human kidney cell line HEK293T were purchased from the ATCC repository in National Centre for Cell Sciences, Pune, India. Cells were cultured in basal medium (Dulbecco’s modified Eagle’s medium and RPMI) supplemented with 10% fetal bovine serum in a humidified incubator with 5% carbon dioxide at 37 °C.

### Microarray data analysis

Publicly available colon cancer dataset GSE14333 was used to investigate colon cancer patient samples. These datasets were downloaded from Gene Expression Omnibus (GEO) which can be accessed at http://www.ncbi.nlm.nih.gov/geo/ and analyzed using GeneSpring GX 12.0 (Agilent Inc., USA). Initially, the probe set IDs representing EVI1, SLUG, SNAIL, ZEB1, ZEB2 TWIST1, TWIST2, E-CADHERIN, N-CADHERIN, and CD44 were identified in each of the datasets. The values for multiple probe sets representing each of our candidate genes were then averaged, and the mean was used to analyze the correlation between EVI1 and the above-mentioned transcription factors in the chosen datasets and plotted.

### Preparation of cell lysate and western blot analysis

Cells were washed with ice-cold Phosphate buffered solution (PBS) and lysed in NP-40 based lysis buffer (Tris-25mM, NaCl-50mM, NP-40 1%, 1X protease inhibitor). Protein samples were resolved on SDS polyacrylamide gel and blotted onto polyvinylidene difluoride (PVDF) membrane (Millipore, USA), and then probed with primary antibodies against EVI1, E-CADHERIN, ACTIN from Cell Signalling Technology, USA. N-CADHERIN (EMD Millipore, USA), SLUG (mouse polyclonal) and HRP-conjugated rabbit/mouse secondary antibodies (Santa Cruz Biotechnology, USA) were used. Western blot analysis was accomplished according to standard procedures using ECL reagent (Thermo Scientific, USA).

### ChIP assay

The ChIP assay was performed using a chromatin immunoprecipitation assay kit (Imgenex, India) according to the manufacturer’s instructions with some modifications. Specific primers were used for amplifying the binding region in human SLUG promoter. Primer sequences (5′-3′)

SLUG-ChIP-F1 AGAAACGCTGTGCTCCAGGCAGATG

SLUG-ChIP-R1 GTAAAACGAGGGGTACCTAGTAGTTC

SLUG-ChIP-F2 CATAAAAGCAGGGGAATATAG

SLUG-ChIP-R2 GTCCGGTGGTTCCAAATGACAG

The amplified products were separated by native PAGE electrophoresis and visualized by ethidium bromide staining.

### Construction of SLUG promoter-luciferase constructs

PCR reactions were carried out with the sequence-specific primer pairs; these primers were designed to contain KpnI and XhoI site for the subsequent cloning reactions. The nomenclature pGL3 (−1200 bp) luc reporter vector is based on the length of the insert, upstream to the transcription start site of the SLUG promoter. Desired DNA fragments were PCR amplified by using forward primer 5′GTCGGTACCTCTGCAATGGACAGAG 3′ and the reverse primer 5′CTGCTCGAGTCAGCTCCTCCCTCTG 3′, and inserted into luciferase reporter vector pGL3-basic (Promega, USA). The inserts were positioned in sense orientation relative to the luciferase coding sequence between KpnI and XhoI. The first binding site was deleted from pGL3-SLUG (−1200 bp) luc vector by digestion to obtain pGL3-SLUG (−950 bp) luc vector. Proper insertion/deletion of both the constructs was verified by direct sequencing.

### Luciferase reporter assay

293T cells were seeded in 6-well tissue culture plates. After 24 h, 1 µg of each of the SLUG luciferase reporter construct was transfected along with either 2 µg of CMV-empty vector or flag-EVI1-wt. For transfection efficiency, the renilla luciferase plasmid DNA (0.5 µg) construct was transfected using calcium chloride precipitation method. Luciferase activity was measured after 24 h using a luciferase assay kit in a luminometer (GLOMAX 20/20, Promega, USA) following the protocol of the manufacturer.

### Real-time quantitative PCR

To knock down, the expression of EVI1, specific EVI1 siRNA (a combination of three siRNA’s) was transfected into COLO205 cells by using the Icafectin siRNA transfection reagent (Eurogentec, Belgium).

EVI1 siRNA sequences:

5′ CCUGCUAGUUCUCCUGUUAdTdT 3′

5′ UAACAGGAGAACUAGCAGG 3′

5′ CCCUGAGGAUGACUAUGAAdTdT 3′

5′ UUCAUAGUCAUCCUCAGGG 3′

5′ GAAUCUGGCUUCAAUAAAUdTdT 3′

5′ AUUUAUUGAAGCCAGAUUC 3′

The Real-Time PCR master mix containing SYBR Green (Roche, Switzerland) was used for Real-Time PCR on Light Cycler 2.0 (Opticon2, MJ Research), and the data was recorded and analyzed. The data was normalized to the endogenous expression of beta-actin.

Primer sequences (5′-3′)

EVI1-F

CTTCTTGACTAAAGCCCTTGGA

EVI1-R

TGTTGGAAGCTGGCTCAAGTAG

SLUG-F

GCCTCCAAAAAGCCAAACTA

SLUG-R

CACAGTGATGGGGCTGTATG

E-CADHERIN-F

GGGCAGAGTGAATTTTGAAG

E-CADHERIN-R

CTTTGGTGGAAAACTTTCTG

N-CADHERIN-F

CTGAGCATGCCAAGTTCCTG

N-CADHERIN-R

CTTTGTAGGTGGCCACTGTG

ACTIN-F

CCTTCCTGGGCATGGAGTCCT

ACTIN-R

GGAGCAATGATCTTGATCTTC

### Transwell migration assay

Matrigel-based migration assay was performed using Corning Biocoat matrigel invasion chamber (Corning, USA). Approximately 3.0 × 10^4^ cells were added to the top chamber of 24 well 8.0 µm transwell inserts, and complete media was added to the bottom chamber as the chemoattractant. After 48 h of incubation, the cells in the top chamber that had failed to migrate were removed, and the cells that migrated to the bottom chamber were taken and cultured.

### Flow cytometry assay

EMT induced COLO205 cells were washed with PBS. Then, cells were incubated on ice for one hour with monoclonal antibodies specific for human cell surface markers including CD44-FITC (Miltenyi Biotech, Germany). In negative control experiments, cells were incubated with fluorescence-labeled isotype-matched pre-immune IgG. The cells were washed and analyzed using a flow cytometer (BD FACS Calibur).

### Animals

NOD SCID mice aged between 6–8 months were used for the experiment. The study was carried out in compliance with the Institutional animal ethics guidelines, and the protocol was approved by the committee (IAEC/276/2013).

### Human colon cancer cell metastatic mouse model

EMT induced COLO205 cells were used for the study. The animals (6–8 week old) were divided into two groups with six animals per group, and 1 × 10^6^ cells in 100 µL of serum-free media was injected into one of the lateral tail vein. Animals were sacrificed after 4 weeks of injection of the cells and necropsies done to identify macro-metastases. The tumors and visceral organs were collected, fixed in neutral buffered formalin and paraffin embedded. 4–5 µm sections were taken and stained using hematoxylin and eosin staining to evaluate the macro and micro-metastases.

### Statistical analysis

Statistical analysis was done with Prism 5.0 (GraphPad Software, Inc. USA). All data are shown as mean ± S.D. We subjected all data greater than two groups to one-way ANOVA. When ANOVA indicated a significant difference, we explored individual differences with two-tailed Student’s t-test. All the correlation data sets were assessed by using Pearson rank correlation test. The criterion for statistical significance was *p* < 0.05.
